# Galactomannan in Bronchoalveolar Lavage Fluid for Diagnosis of Invasive Pulmonary Aspergillosis with Nonneutropenic Patients

**DOI:** 10.1155/2017/3685261

**Published:** 2017-11-13

**Authors:** Qidong Zhuang, Hongying Ma, Yun Zhang, Lei Chen, Li Wang, Lin Zheng, Zaichun Deng, Zhongbo Chen

**Affiliations:** ^1^Department of Pulmonology, The Affiliated Hospital of Ningbo University, 247 Renmin Road, Ningbo, Zhejiang 315020, China; ^2^Department of Microbiology, The Affiliated Hospital of Ningbo University, 247 Renmin Road, Ningbo, Zhejiang 315020, China

## Abstract

**Background:**

We evaluated the utility of galactomannan (GM) in bronchoalveolar lavage fluid (BALF) for the diagnosis of invasive pulmonary aspergillosis (IPA) in nonneutropenic patients.

**Methods:**

A total of 183 patients were included in the final analysis. Bronchoscopies and the detection of GM in BALF were all performed on them.

**Results:**

Ten cases of IPA were diagnosed. ROC data demonstrated that, for diagnosing IPA, an optimal cutoff value for GM in BALF of 0.76 yielded a sensitivity of 100.0% and a specificity of 76.2%. Symptoms and radiological findings had no significant difference between proven or probable IPA group and non-IPA group. In our case-control analysis, although nine patients with false-positive results received treatment with Piperacillin/tazobactam, there was no significant difference between case and control group.

**Conclusions:**

BALF GM detection is a valuable adjunctive diagnostic tool. Our retrospective study suggests that the optimal value of GM detection in BALF is 0.76 in nonneutropenic patients.

## 1. Introduction

*Aspergillus* is a ubiquitous fungus which can cause various kinds of pulmonary aspergillosis, such as allergic bronchopulmonary aspergillosis (ABPA), pulmonary aspergilloma (PA), and invasive pulmonary aspergillosis [1] (IPA). IPA is the most severe type in the pulmonary aspergillosis. Moreover, it is an important cause of fatality in immunocompromised populations, especially in hematological and solid-organ transplant patients [2]. Once a study reported that the morbidity of IPA might be 15% to 20% in HSCT recipients and neutropenic patients with hematologic malignancies, and the mortality can be as high as 50% to 90% [3]. In the past, neutropenia was considered a major risk for IPA. However, recent studies reported neutropenia was absent in a number of IPA patients [4, 5]. In nonneutropenic patients, chronic obstructive pulmonary disease (COPD) was the most common underlying disease [4]. Besides, 19.2%–22.2% patients with IPA had no diseases, whose fatality rate was as high as 33.3% to 39%. Current evidence suggests that antifungal therapy should be initiated as early as possible to improve outcome. So early detection, timely diagnosis, and early treatment are the keys to decrease the mortality.

As the development of chest computer tomography, a variety of pulmonary diseases can be diagnosed. The classic halo sign and air crescent sign are frequent in neutropenic hosts with IPA. However, the above signs are rare and less specific among nonneutropenic patients [6]. Moreover, pulmonary consolidation, infiltration, and nodular lesions are common. The current gold standard for the diagnosis of proven IPA requires histological evidence. But the invasive procedures carry high risks for the patients with underlying diseases. The microbiological culture is time-consuming and insensitive [7–9]. So it is urgent for us to seek a new method to detect the* Aspergillus*.

Galactomannan (GM) is a polysaccharide component of the cell wall of* Aspergillus* [7], which can be released into the body fluids by growing hyphae, such as blood, urine, and bronchoalveolar fluid (BALF). With using in vitro and animal models of IPA, the researchers suggested that GM might be released at an earlier stage and at a higher concentration into the BALF than in serum [8]. Currently, serum GM assay has been used for supervising, early detection, and timely diagnosis [10] in neutropenic hosts. However, the diagnostic value of BALF GM assay for IPA is still uncertain, especially in the nonneutropenic patients. To further investigate the utility of GM in BALF for the diagnosis of IPA in nonneutropenic hosts, we conducted this retrospective study.

## 2. Methods

### 2.1. Study Population

All nonneutropenic patients coming from Affiliated Hospital of Ningbo University, who were admitted to Respiratory Medicine Department from April 2014 to February 2017, were reviewed. Written informed consent was obtained from each patient. And the Institutional Review Board approved this work.

### 2.2. Bronchoscopy and Sample Collecting

Experienced doctors performed all bronchoscopies with bronchoalveolar lavage (20 ml for three times). The site of bronchoscopy was guided by the lung CT scan at first. Based on the infiltration location on the chest radiograph, the sampling area was selected. If there were diffuse lesions in bilateral lungs, the bronchoscope would be wedged into the middle lobe. If there were multiple lesions in bilateral lungs, the bronchoscope would be wedged into the most serious segment of left and right lung, respectively; then the lavage samples were mixed. If there were multiple lesions in unilateral lung, the bronchoscope would be wedged into the most serious segment or the involved segments and then the lavage samples mixed.

The lavage samples were submitted for direct microscopic examination and microbiological culture. Besides, a vortex was done for the remaining BALF samples and the supernatant was used for GM detection by using the Platelia Aspergillus EIA (Bio-Rad). An OD index ≧ 0.8 for BALF GM was considered positive [2, 11, 12].

### 2.3. Case Definitions

Each of the included IPA patients was classified based on the criteria of the European Organization for Research and Treatment of Cancer and the Mycoses Study Group (EORTC/MSG) revised in 2008. Proven cases required histopathological evidence. Probable cases required at least a host factor, a radiological criterion, and a kind of microbiological evidence. Possible cases only required hosts and radiological criteria without microbiological proofs.

Receipt of an allogeneic stem cell transplantation, prolonged use of corticosteroids, hematological diseases, solid-organ transplantation, and treatment with T cell immunosuppressants were classic risk factors. In recent years, critically ill patients with COPD [2] and patients with cirrhosis [13] were also host factors. Moreover, diabetes mellitus, rheumatologic disease, and bronchiectasis had been reported as the underlying diseases for IPA [4]. Patients with above diseases were considered as host factors, except for neutropenic patients. Because the imaging manifestations were less specific for nonneutropenic groups with IPA, pulmonary consolidation, infiltration, or nodular lesions were considered the radiological criteria. Based on previous studies [14–16], the modified microbiological criteria included any of the following: (1) positive isolation of* Aspergillus* species from sputum or BALF by culture; (2) a positive serum GM result (ODI ≧ 0.5) verified in two consecutive samples; (3) a positive BALF GM result (ODI ≧ 0.8) with, simultaneously, antibiotic treatment invalid but antifungal treatment against aspergillosis valid.

The “non-IPA” patients included those with ABPA, aspergilloma, and the colonization or contamination, except for the IPA patients. To compare the characteristics of IPA patients with that of non-IPA ones, patients in whom there was no evidence of IPA were classified as the “non-IPA” group, which excluded ABPA, aspergilloma, and the colonization or contamination group. Colonization or contamination group was defined as positive* Aspergillus* culture from sputum or BALF, who clinically improved without antifungal therapy.

### 2.4. Case-Control Analysis

To analyze the influences of different risk factors on BALF GM detection, non-IPA patients were divided into two groups: patients with positive BALF GM results (case patients) and patients with negative BALF GM results (control patients). Both groups excluded the IPA, ABPA, aspergilloma, and the colonization or contamination patients.

### 2.5. Statistical Analysis

BALF GM assay should be calculated sensitivity, specificity, positive predictive index (PPV), and negative predictive index (NPV). Although we considered an ODI ratio ≧ 0.8 in BALF GM as the positive result for referenced diagnosis of IPA, the receiver operating characteristic (ROC) curve was used to acquire the optimal cutoff index. Furthermore, factors causing false-positive results in the BALF GM assay were analyzed. Quantitative variables were compared using *t*-tests. For qualitative date, Pearson's *χ*^2^ or Fisher's exact tests were used. Logistic regression analysis was performed to identify risk factors for false-positive BALF GM results. All tests used were two-tailed, and a *p* value of <0.05 was considered statistically significant.

## 3. Results

### 3.1. Patient Characteristics

**Figure 1:**
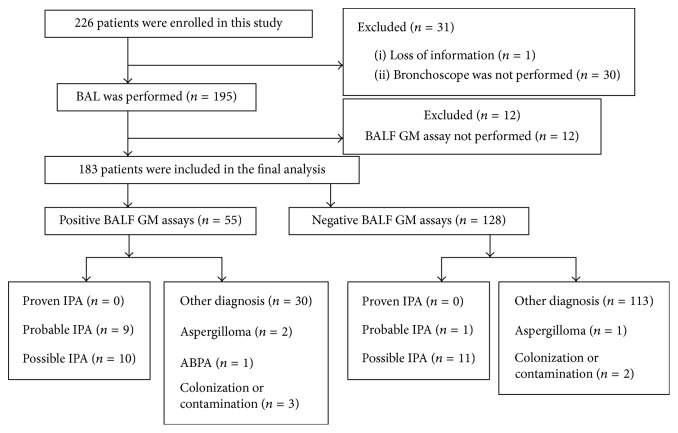
Study flow diagram. BAL, bronchoalveolar lavage; BALF, bronchoalveolar lavage fluid; GM, galactomannan; IPA, invasive pulmonary aspergillosis; ABPA, allergic bronchopulmonary aspergillosis.

During the study period, a total of 226 inpatients were enrolled in. Of those, 43 patients were excluded from the study, including 30 patients without the performance of bronchoscopy, 1 patient with lack clinical information, and 12 patients without undergoing the BALF GM assay. Thus, 183 patients participated in our final analysis (Figure 1). The baseline clinical characteristics of chosen patients with IPA and “non-IPA” groups are shown in Table 1. IPA patients were classified as proven IPA (*n* = 0), probable IPA (*n* = 10), and possible IPA (*n* = 21). Non-IPA patients included non-IPA (*n* = 143), ABPA (*n* = 1), aspergilloma (*n* = 3), and colonization or contamination hosts (*n* = 5).

In the proven, probable, and possible IPA patients, the pulmonary diseases were the main underlying condition, such as emphysema (*n* = 5), COPD (*n* = 1), pulmonary tuberculosis (*n* = 2), bronchiectasis (*n* = 3), and lung cancer (*n* = 3). Nine (29.0%) patients had no underlying diseases.

### 3.2. Clinical Symptoms and Radiological Findings

Table 2 shows the prominent symptoms and radiological findings in patients with proven or probable IPA. Each patient complaint about at least one of following symptoms: cough (*n* = 9, 90.0%), dyspnoea (*n* = 3, 30.0%), fever (*n* = 2, 20.0%), haemoptysis (*n* = 2, 20.0%), chest tightness (*n* = 1, 10.0%), and chest pain (*n* = 1, 10.0%). Chest computed tomography (CT) was performed in all included patients. The most frequent abnormality was infiltrate or nodules (*n* = 8, 80.0%). Common imaging findings occurring in neutropenic patients, such as halo sign and air crescent sign, were observed in only one patient. However, there was no significant difference between proven or probable IPA group and non-IPA group. Besides, there was also no significant difference in seasonal distribution.

### 3.3. Performance of BALF GM Assay

**Figure 2:**
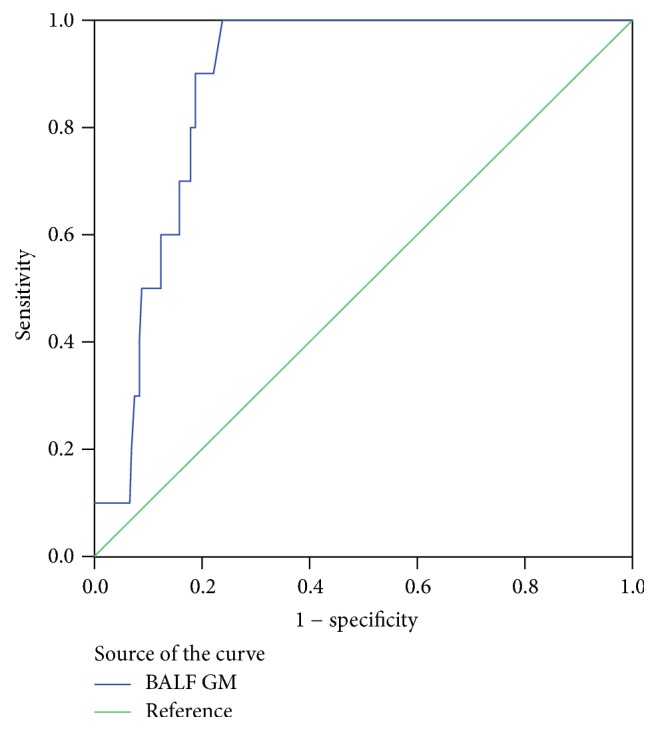
Receiver operating characteristic (ROC) curves for galactomannan assay in 183 study populations. Areas under the ROC curve was 0.88 (95% CI 0.82–0.94).

The sensitivity, specificity, PPV, and NPV of BALF GM assay are shown in Table 3. We considered proven and probable IPA as true-positives. When the OD index cutoff to define BALF GM positivity was lowered from ≥2.0 to ≥0.5, the estimated sensitivity increased from 50.0% to 100.0%, and the specificity decreased from 88.8% to 64.3%. At the index cutoff value of ≥0.8, the BALF GM assay had a sensitivity of 90% and a specificity of 78.3%. ROC data demonstrated that, for diagnosing IPA, an optimal cutoff value for GM in BALF of 0.76 yielded a sensitivity of 100.0% and a specificity of 76.2%, and that the diagnostic accuracy of BALF GM as determined by the area under the ROC curve was 0.88 (95% CI 0.82–0.94) (Figure 2).

### 3.4. Case-Control Analysis

The positive BALF GM assay result occurred in 55 patients, 9 were diagnosed as probable IPA, 10 were classified as possible IPA, 1 was considered as ABPA, 2 were diagnosed as aspergilloma, 3 were defined as colonization or contamination, and the remaining 30 were regarded as other diagnoses. To assess the factors of BALF GM assay with false-positive results, we performed a case-control analysis. Univariate and multivariate analysis of the date are shown in Table 4. Nine patients with false-positive results were treated with Piperacillin/tazobactam, but there was no significant difference between the case and control group.

## 4. Discussion

IPA is a life-threatening disease, especially in immunocompromised hosts. The classic risk factors for IPA include neutropenia, receipt of an allogeneic stem cell transplant, long-time use of corticosteroids and treatment with immunosuppressants [10]. With the in-depth studies, hematologic diseases [9, 17], COPD [18], liver cirrhosis [19], and intensive care unit patients [20] are also susceptible individuals. Furthermore, in recent researches [4, 5], organ failure, pulmonary tuberculosis, bronchiectasis, and diabetes patients and even those with no underlying diseases can be infected by* Aspergillus*, which is similar to our study. Notably, in our small sample, 29.0% of 31 probable/possible IPA patients were void of underlying diseases. Besides, the pulmonary diseases were the most common underlying condition. In patients with airways or lung parenchyma damaged by underlying respiratory diseases, the defense function is limited. When they inhale the* Aspergillus* into the respiratory tract, the* Aspergillus* may colonize and even cause IPA. To find out the specific clinical symptoms and radiological presentation, we compared proven/probable IPA patients with non-IPA individuals. Consequently, as previous studies recorded [6, 9], the classic halo sign and air crescent sign were far less common and specific in nonneutropenic ones. Furthermore, there were no significant differences in frequency of various symptoms or imaging findings.

Rapid initiation of systemic antifungal treatment is the key to improve the prognosis of IPA patients. Diagnosis of IPA is always an arduous task, although a large number of reports have demonstrated that the detection of GM in blood samples coming from neutropenic patients is helpful for early diagnosis. However, the utility of BALF GM assay still needs to be investigated by many clinical studies, especially in nonneutropenic hosts. Based on the previous findings [2, 3, 7–9, 17, 18], the optimal cutoff value of BALF GM assay fluctuated, ranging from 0.5 to 1.25 in patients with different underlying diseases. A meta-analysis [19] of detecting galactomannan in BALF suggested the optimal cutoff value was 1.0. In our study, we evaluated the efficacy of measuring GM concentration in BALF for diagnosing IPA in nonneutropenic patients. According to the ROC data, the optimal cutoff index was 0.76, and the sensitivity and specificity were 100.0% and 76.2% for diagnosing IPA, respectively. This cutoff value was consistent with the result of other researches [2, 12]. Besides, as previous studies reported, the GM detection of BALF samples had high negative predictive value. In our finding, although the cutoff value ranged from 0.5 to 2.0, the negative predictive value was always over 96.2%.

Various factors can cause false-positive result in BALF GM detection, such as BALF sample pretreatment with Sputasol [21], antifungal treatment, and *β*-lactam antibiotics. *β*-Lactam antibiotics, especially Piperacillin/tazobactam and Mezlocillin/sulbactam, were usually used in patients with bacterial pneumonia. As they are semisynthetic drugs derived from natural compounds produced by other molds, many false-positive BALF GM results from patients taking them have been reported [22]. However, in other studies [14, 18], BALF GM false-positive rates were not significantly different between patients treated with *β*-lactam antibiotics and those who did not receive these drugs. In our study, although 11 non-IPA patients treated with Piperacillin/tazobactam and Mezlocillin/sulbactam had false-positive results in BALF GM detection, there was no significant difference between the case and control group. Colonization or contamination of the* Aspergillus* in the respiratory tract may be another reason for false-positive results. We should note that 3 patients considered as colonization or contamination had false-positive values in BALF GM assay. In spite of negative culture results, the patients with underlying pulmonary diseases may be colonized by* Aspergillus*, which could be another contributing factor for false positivity.

This study has several limitations. First, the present study was retrospective. We may perform a prospective research in the future. Secondly, the number of patients with proven IPA was small; in fact, none of them had proven IPA. Besides, the patients came from a single center. So we will enlarge the number of target population and invite other hospitals to participate in the following research. Thirdly, we used GM detection in BALF as part of mycological criterion for diagnosing, which may cause incorporation bias. If the results of BALF GM were excluded from mycological criteria of IPA, 3 of 10 probable IPA patients were reclassified as possible cases. The sensitivity of BALF GM assay may be higher than it really is.

In conclusion, our retrospective study suggests that the optimal value of GM detection in BALF is 0.76 in nonneutropenic patients. However, the utility of GM in BALF for diagnosing IPA in nonneutropenic hosts still needs to be evaluated by lots of multicenter studies.

## Figures and Tables

**Table 1:** Baseline clinical characteristics of the study population^a^.

	IPA (*n* = 31), *n* (%)	Non-IPA (*n* = 152), *n* (%)
Age, mean years	58.97	55.62
Male gender	20 (64.5)	82 (53.9)
High-risk environment exposed	2 (6.5)	1 (0.7)
Underlying disease		
Emphysema	5 (16.1)	22 (14.5)
COPD	1 (3.2)	8 (5.3)
Bronchial asthma	0	8 (5.3)
Pulmonary tuberculosis	2 (6.5)	10 (6.6)
Solid tumor	3 (9.7)	8 (5.3)
Bronchiectasis	3 (9.7)	24 (15.8)
Diabetes	5 (16.1)	16 (10.5)
Liver cirrhosis	0	1 (0.7)
Hematologic malignancy	0	1 (0.7)
Autoimmune disease	2 (6.5)	2 (1.3)
Kidney non-malignant disease	0	3 (2.0)
Organ failure	2 (6.5)	1 (0.7)
No underlying disease	9 (29.0)	57 (37.5)
Final diagnosis		
	Proven: 0	ABPA: 1 (0.7)
	Probable: 10 (32.3)	Aspergilloma: 3 (2.0)
	Possible: 21 (67.7)	Colonization or contamination: 5 (3.3)
		Others: 143 (94.1)

IPA, invasive pulmonary aspergillosis; COPD, chronic obstructive pulmonary disease; ABPA, allergic bronchopulmonary aspergillosis. ^a^Date are number (%) of patients, unless otherwise indicated.

**Table 2:** Comparison of seasonal distribution, clinical symptoms, and chest CT between proven/probable IPA patients and non-IPA patients.

Characteristics	Proven/probable IPA (*n* = 10)	Non-IPA^a^ (*n* = 143)	*p*
Age ≥ 60 years	6 (60.0)	63 (44.1 )	0.515
Male gender	8 (80.0)	77 (53.8)	0.201
Seasonal distribution, *n* (%)			
March–October	7 (70.0)	93 (65.0)	1.000
Clinical symptoms, *n* (%)			
Cough	9 (90.0)	101 (70.6)	0.340
Fever	2 (20.0)	37 (25.9)	0.971
Dyspnoea	3 (30.0)	16 (11.2)	0.212
Haemoptysis	2 (20.0)	21 (14.7)	1.000
Chest tightness	1 (10.0)	12 (8.4)	0.600
Chest pain	1 (10.0)	18 (12.6)	1.000
Chest CT, *n* (%)			
Infiltrate/Nodules	8 (80.0)	100 (69.9)	0.751
Bronchiectasis	3 (30.0)	28 (19.6)	0.700
Cavity	3 (30.0)	18 (12.6)	0.284
Air crescent sign	1 (10.0)	0	0.065
Halo sign	1 (10.0)	10 (7.0)	0.537
Other	1 (10.0)	12 (8.4)	0.600

IPA, invasive pulmonary aspergillosis; CT, computer tomography. ^a^Non-IPA patients are defined as individuals in the “non-IPA” group excluding aspergilloma, ABPA, colonization, or contamination patients.

**Table 3:** Performance of GM detection for diagnosis of IPA in BALF.

Cutoff value	Sensitivity%	Specificity%	PPV%	NPV%
BALF GM ≥ 0.5	100.0%	64.3%	16.4%	100.0%
BALF GM ≥ 0.76	100.0%	76.2%	22.7%	100.0%
BALF GM ≥ 0.8	90.0%	78.3%	22.5%	99.1%
BALF GM ≥ 1.0	70.0%	82.5%	21.9%	97.5%
BALF GM ≥ 1.5	60.0%	86.0%	23.1%	96.8%
BALF GM ≥ 2.0	50.0%	88.8%	23.8%	96.2%

GM, galactomannan; IPA, invasive pulmonary aspergillosis; BALF, bronchoalveolar lavage fluid; PPV, positive predictive value; NPV, negative predictive value.

**Table 4:** Risk factors for false-positive galactomannan results in bronchoalveolar lavage assays with univariate analysis and logistic regression analysis, respectively.

Variables	Case patients^a^ (*n* = 30), *n* (%)	Control patients^b^ (*n* = 113), *n* (%)	*p*	
			Univariate analysis	Logistic regression analysis
Age ≥ 60 years	12 (40.0)	51 (45.1)	0.615	0.533
Male gender	13 (43.3)	64 (56.6)	0.194	0.067
Seasonal distribution				
March–October	23 (76.7)	70 (61.9)	0.133	0.129
Underlying disease				
Emphysema	2 (6.7)	10 (8.8)	0.990	0.608
COPD	3 (10.0)	5 (4.4)	0.463	0.121
Bronchial asthma	2 (6.7)	5 (4.4)	0.976	0.273
Pulmonary tuberculosis	7 (23.3)	10 (8.8)	0.063	0.117
Solid tumor	0	11 (9.7)	0.164	0.999
Bronchiectasis	7 (23.3)	16 (14.2)	0.349	0.431
Diabetes	0	11 (9.7)	0.164	0.999
Liver cirrhosis	0	1 (0.9)	1.000	0.999
Hematologic malignancy	0	1 (0.9)	1.000	1.000
Autoimmune disease	0	2 (1.8)	1.000	0.999
Kidney non-malignant disease	1 (3.3)	1 (0.9)	0.377	1.000
Antibiotics				
Piperacillin/tazobactam	9 (30.0)	22 (19.5)	0.213	0.479
Mezlocillin/sulbactam	2 (6.7)	5 (4.4)	0.976	0.726
Cephalosporins	7 (23.3)	49 (43.4)	0.046	0.157
Quinolones	7 (23.3)	37 (32.7)	0.321	0.404

COPD, chronic obstructive pulmonary disease. ^a^Case patients are defined as individuals in the “non-invasive pulmonary aspergillosis (IPA)” group with positive bronchoalveolar lavage fluid (BALF) galactomannan (GM) results, excluding aspergilloma, ABPA, colonization, or contamination. Namely, the group is the other diagnosis with positive BALF GM results. ^b^Control patients are defined as individuals in the “non-IPA” group with negative BALF GM results, excluding aspergilloma, colonization, or contamination. Namely, the group is the other diagnosis with negative BALF GM results.
